# *Plasmodium vivax* Population Structure and Transmission Dynamics in Sabah Malaysia

**DOI:** 10.1371/journal.pone.0082553

**Published:** 2013-12-17

**Authors:** Noor Rain Abdullah, Bridget E. Barber, Timothy William, Nor Azrina Norahmad, Umi Rubiah Satsu, Prem Kumar Muniandy, Zakiah Ismail, Matthew J. Grigg, Jenarun Jelip, Kim Piera, Lorenz von Seidlein, Tsin W. Yeo, Nicholas M. Anstey, Ric N. Price, Sarah Auburn

**Affiliations:** 1 Herbal Medicine Research Centre, Institute for Medical Research, Kuala Lumpar, Malaysia; 2 Infectious Diseases Unit, Queen Elizabeth Hospital, Kota Kinabalu, Sabah, Malaysia; 3 Global and Tropical Health Division, Menzies School of Health Research and Charles Darwin University, Darwin, Australia; 4 Sabah Department of Health, Kota Kinabalu, Sabah, Malaysia; 5 Division of Medicine, Royal Darwin Hospital, Darwin, Australia; 6 Centre for Tropical Medicine, Nuffield Department of Clinical Medicine, University of Oxford, Oxford, United Kingdom; Universidade Federal de Minas Gerais, Brazil

## Abstract

Despite significant progress in the control of malaria in Malaysia, the complex transmission dynamics of *P. vivax* continue to challenge national efforts to achieve elimination. To assess the impact of ongoing interventions on *P. vivax* transmission dynamics in Sabah, we genotyped 9 short tandem repeat markers in a total of 97 isolates (8 recurrences) from across Sabah, with a focus on two districts, Kota Marudu (KM, n = 24) and Kota Kinabalu (KK, n = 21), over a 2 year period. STRUCTURE analysis on the Sabah-wide dataset demonstrated multiple sub-populations. Significant differentiation (*F*_ST_  = 0.243) was observed between KM and KK, located just 130 Km apart. Consistent with low endemic transmission, infection complexity was modest in both KM (mean MOI  = 1.38) and KK (mean MOI  = 1.19). However, population diversity remained moderate (*H*_E_  = 0.583 in KM and *H*_E_  = 0.667 in KK). Temporal trends revealed clonal expansions reflecting epidemic transmission dynamics. The haplotypes of these isolates declined in frequency over time, but persisted at low frequency throughout the study duration. A diverse array of low frequency isolates were detected in both KM and KK, some likely reflecting remnants of previous expansions. In accordance with clonal expansions, high levels of Linkage Disequilibrium (*I*_A_^S^ >0.5 [*P*<0.0001] in KK and KM) declined sharply when identical haplotypes were represented once (*I*_A_^S^  = 0.07 [*P* = 0.0076] in KM, and *I*_A_^S^ = -0.003 [*P* = 0.606] in KK). All 8 recurrences, likely to be relapses, were homologous to the prior infection. These recurrences may promote the persistence of parasite lineages, sustaining local diversity. In summary, Sabah's shrinking *P. vivax* population appears to have rendered this low endemic setting vulnerable to epidemic expansions. Migration may play an important role in the introduction of new parasite strains leading to epidemic expansions, with important implications for malaria elimination.

## Background

With an estimated 2.85 billion people living at risk of infection [1], *Plasmodium vivax* is the most widely distributed of the *Plasmodium* species infecting humans. Accumulating reports of resistance to chloroquine and associated severe and fatal disease complications [2] demonstrate the importance of addressing the particular challenges faced in controlling and eliminating this parasite. *P. vivax* differs from *P. falciparum* in having a dormant hypnozoite stage, greater asymptomatic asexual carriage and early gametocyte production, these factors enhancing the parasite's potential for local transmission. For this reason, the elimination of *P. vivax* faces far greater obstacles than that of *P. falciparum* [3], [4]. With concerted efforts to eliminate *P. falciparum* and resultant decline in prevalence, *P. vivax* is becoming the dominant species of malaria in many coendemic regions.

Malaysia has demonstrated its commitment to the control and elimination of malaria, with national programs dating back to the 1960s. There has been a substantial decline in annual cases from an estimated 181,495 in 1967 to 55,000 in 1990 and 6,426 in 2010 [5]. Malaria control efforts have been particularly effective in Peninsular Malaysia, whilst the remote regions of Sabah and Sarawak, on the island of Borneo, have proven more challenging owing to constraints in accessibility to effective diagnostics and treatment. Malaysia is now committed to eliminating malaria by 2015 in the Peninsular region and 2020 on the island of Borneo [5].

Four species of malaria (*P. falciparum*, *P. vivax, P. malariae and P. knowlesi*) are endemic in Malaysia, with *P. vivax* contributing to more than 50% of the malaria cases nationwide in 2010 [5]. However, exact proportions remain uncertain owing to discrepancies between microscopy and PCR-based diagnoses [6], [7]. Recent data indicates that the region most widely affected by malaria is Sabah, with an estimated 24.5% of the population living at risk of infection [8]. In 2011, *P. vivax* accounted for 31% of all malaria notifications [9]. Sabah is located in the north of Borneo, sharing borders with the Malaysian state Sarawak in the south-west, Kalimantan (Indonesia) in the south-east, and the islands of the Philippines to the north. *P. vivax* transmission persists in all of these border regions [5]. In this context, imported malaria is a major threat to elimination efforts, potentially undermining local intervention strategies, enhancing the parasite population diversity and adaptation potential, and increasing the risk of drug resistance spread and outbreaks in host populations with insufficient immunity.

An assessment of *P. falciparum* diversity in Sabah undertaken in 2005 demonstrated a fragmented population structure associated with the declining parasite endemicity [10]. However, the impact on the transmission dynamics and subsequent structure and diversity of the local *P. vivax* population is unknown.

The Asia Pacific Malaria Elimination Network (APMEN) was established in 2009 to address the specific challenges of malaria elimination in the region, including a high prevalence of *P. vivax* infections and the burden of imported malaria [11]. The APMEN Vivax Working Group (VxWG) is a body of 14 country partners that have acknowledged the utility of genotyping to inform on the parasite's complex transmission dynamics and patterns of spread within and across borders, developing a consensus with which to compare and contrast parasite population from a variety of locations. The protocol proposes nine short tandem repeat markers including eight putatively neutral microsatellites (MS1, MS5, MS8, MS10, MS12, MS16, MS20, Pv3.27) and one variable surface antigen marker (msp1F3) [12], [13], [14]. In order to assess the impact of current malaria intervention strategies on the local *P. vivax* transmission dynamics, *P. vivax* isolates from individuals residing in several regions of Sabah were genotyped using the consensus marker set. The resultant data were used to gauge the parasite diversity, population structure and transmission dynamics in the region.

## Materials and Methods

### Ethics

All samples were collected with written informed consent from the patient, parent or legal guardian (individuals <18 years of age). The study was approved by the Research Review Committee of the Institute for Medical Research and the Medical Research Ethics Committee (MREC), Ministry of Health Malaysia and the Human Research Ethics Committee of the NT Department of Health & Families and Menzies School of Health Research, Darwin, Australia.

### Study Sites and sample collection

**Figure 1:**
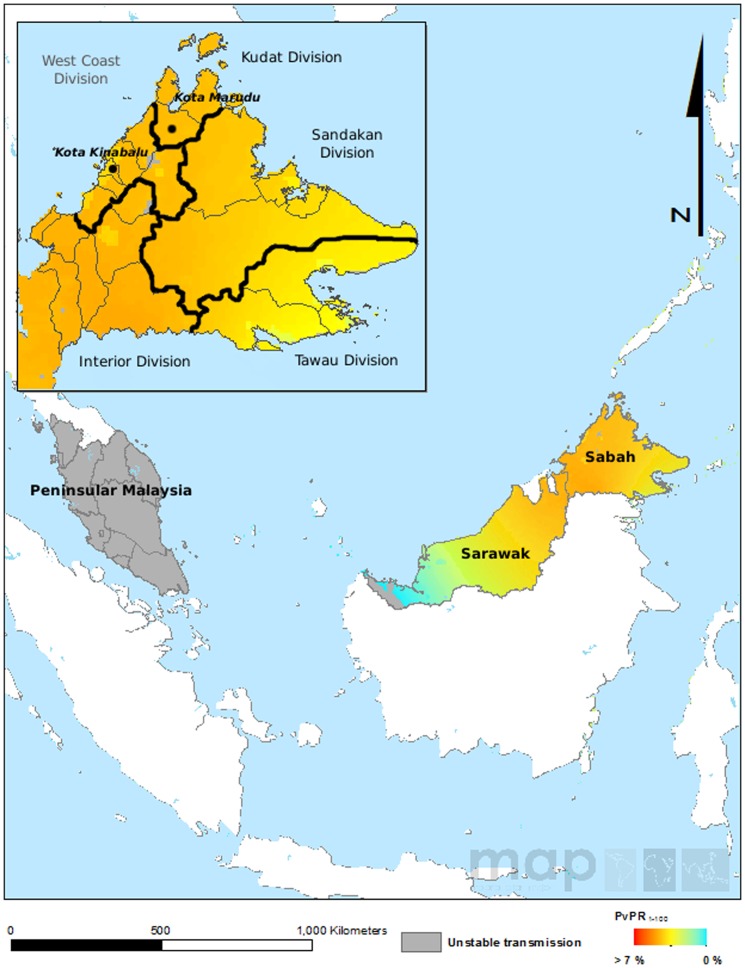
Spatial distribution of *P. vivax* endemicity in 2010 in Malaysia. This map was generated by Zhi Huang, Malaria Atlas Project, University of Oxford. The colour scale reflects the age-standardized *P. vivax* parasite rate (PvPR), which describes the estimated proportion of the general population that are infected with *P. vivax* at any one time, averaged over the 12 months of 2010 within the spatial limits of stable transmission [15]. In this study, samples were collected from patients residing in all 5 of the Sabah divisions (illustrated in the square in the top left corner), but predominantly from Kota Marudu district in the Kudat Division and Kota Kinabalu district in the West Coast Division.

Sabah is divided into 5 administrative divisions (Kudat, Interior, Sandakan, Tawau and West Coast), with heterogeneity observed in the estimated prevalence of endemic *P. vivax* malaria [15]. Kudat Division spans an area of 4,623 square kilometres split between three districts, Kudat, Kota Marudu and Pitas. The West Coast Division covers an area of 7,588 square kilometres, divided into the districts of Ranau, Kota Belud, Tuaran, Penampang, Putatan, Papar, and the state capital Kota Kinabalu. The current study focused on Kota Kinabalu and Kota Marudu districts, where established collaborations with local health researchers at major hospitals and health centers enabled representative sampling from patients in the local districts. These sites, located approximately 130 Km apart, represent low (Kota Marudu; incidence  = 1 case per 1000 population in 2010) and very low (Kota Kinabalu; incidence  = 0.2 cases per 1000 population in 2010) *P. vivax* endemic settings (Figure 1). Kota Marudu district spans a region of approximately 1,917 square kilometres, with an estimated population size of 80,900 in 2010 [16]. Kota Kinabalu district is home to the capital of Sabah, one of the main industrial and commercial centres in East Malaysia. The Malaysian Census 2010 Report estimated a population of 452,058 inhabitants in Kota Kinabalu [16]. Malaria is not markedly seasonal in these regions, which experience a typical equatorial climate with relatively constant temperature and high humidity year round.

With the exception of Tawau, *P. vivax-*infected individuals were recruited by passive case detection of patients presenting at Queen Elizabeth Hospital (QEH) [17], Kota Kinabalu, Kudat District Hospital, or Kota Marudu District Hospital between September 2010 and February 2013. Active case detection was undertaken in villages, palm plantation and logging camps in Tawau between June 2008 and November 2009.

Capillary or venous blood samples were collected from patients with a positive rapid diagnostic test (RDT) (Paramax-3, Zephyr Biomedicals, India) during active case detection, or *Plasmodium spp*. positive thick blood smears on microscopy during passive case detection. Capillary sampling involved spotting ∼200 µl blood onto an FTA card (Whatman), and storage as per the manufacturer's instructions. Venous blood sampling involved withdrawal of 2–5 ml blood into an EDTA-containing tube followed by transfer of a ∼200 µl aliquot to an Eppendorf tube, and storage at −20°C or −80°C until DNA extraction.

Clinical management was based on microscopic diagnosis, in accordance with the national treatment policy for uncomplicated malaria: chloroquine (3 doses, 25 mg/kg body weight) and a 14 day course of unsupervised primaquine (0.5 mg/kg body weight daily) for patients with *P. vivax* monoinfection. Although patients were not prospectively followed after treatment, patients representing to the same clinic within the study period could be matched with their preceding history of malaria.

### DNA extraction and PCR-based species identification

DNA extraction was undertaken on 200 µl samples using Qiagen's QIAamp DNA Blood mini kit following the manufacturer's instructions, with an elution volume of 200 µl. *Plasmodium* species was confirmed by PCR. Detection for *P. vivax, P. falciparum, P. malaria* and *P. ovale* parasites was undertaken using a version of Padley *et al.* [18] with the modification that each species was diagnosed in a separate (non-multiplex) assay. Detection for *P. knowlesi* parasites was undertaken using the method of Imwong *et al.* [19].

### Genotyping

The MS1, MS5, MS8, MS10, MS12 and MS20 assays involved a single round of PCR using a slightly modified version of Karunaweera *et al*. [20]: 6.4 pmol of each primer, 0.1 mM each dNTP (Bioline), 1× PCR buffer, 2.5 mM MgCl_2_, 1 Unit *Taq* DNA polymerase (Qiagen) and 2 µl genomic DNA in a final reaction volume of 20 µl. Thermocycling involved an initial denaturation at 94°C for 2 min, followed by 40 cycles of denaturation at 94°C for 30 sec, annealing at 58°C for 40 sec, elongation at 72°C for 30 sec, followed by a final elongation at 72°C for 5 min.

The MS16, Pv3.27 and msp1F3 assays involved nested and semi-nested PCRs as described by Koepfli *et al.* [14]. PCRs were performed in 20 µl reactions containing 0.25 µM of each primer, 2 µl of 10 x buffer, 0.2 mM each dNTP (Bioline), 2 mM MgCl_2_, 1.5 Unit *Taq* DNA polymerase (Qiagen) and 1 µl genomic DNA. Thermocycling conditions included an initial denaturation at 95°C for 5 min, followed by 30 cycles of denaturation at 95°C for 1 min, annealing (56°C for MS16; 58°C for Pv3.27; 59°C for msp1F3) for 1 min, elongation at 72°C for 1 min, followed by a final elongation at 72°C for 5 min. PCR products were diluted as necessary, and 1 µl of diluted product was used as template for the nested (MS16, msp1F3) or semi-nested (Pv3.27) assay, in a 20 µl volume with reagent concentrations as for the primary reaction. Thermocycling conditions for the nest reaction included an initial denaturation at 95°C for 5 min, followed by 25 cycles of denaturation at 95°C for 1 min, annealing (57°C for MS16; 58°C for Pv3.27; 60°C for msp1F3) for 1 min, elongation at 72°C for 1 min, followed by a final elongation at 72°C for 5 min.

The final PCR products were diluted as necessary and sized by denaturing capillary electrophoresis on an ABI 3130xl Genetic Analyzer (Applied Biosystems, Foster City, CA) with GeneScan LIZ-600 (Applied Biosystems) internal size standards. Genotype calling was facilitated with GeneMapper version 4.0. software (Applied Biosystems). A minimum arbitrary fluorescence intensity of 100 fluorescence units was set to exclude potential false peaks arising from background ‘noise’. All electropherogram traces were additionally inspected manually. For each isolate, at each locus, the predominant allele (maximum height peak), and any additional alleles with peak height ≥33% the height of the predominant allele were scored. The implementation of a peak intensity threshold facilitates comparability between samples and populations by reducing potential bias in polyclonality estimates arising from differences in sample DNA quantity [21]. Genotyping success was defined as the presence of at least one allele at a given locus in a given sample.

### Population Genetic Analysis

An infection was defined as polyclonal if two or more alleles were observed at one or more of the 9 loci investigated. The Multiplicity of Infection (MOI) for a given sample was defined as the maximum number of alleles observed at any of the 9 loci investigated. The average MOI was calculated by dividing the sum of MOIs across all samples by the number of samples.

‘Infection haplotypes’ were reconstructed from the predominant allele at each locus, as in other studies [21], [22], [23]. Population-level genetic diversity was characterised using a measure of the expected heterozygosity (*H*_E_). Only the predominant allele at each locus in each isolate was used to ensure an unbiased estimate of the minor allele frequency [21]. *H*_E_ provides a measure of the probability that two unrelated parasites will exhibit different genotypes at a given locus. *H*_E_ was calculated for each locus in each population using the following formula: *H*_E_  = [*n*/(*n*−1)][1−Σ*p* ^2^ *i*], where *n* is the number of isolates analyzed and *pi* is the frequency of the *ith* allele in the population. The inclusion of ‘*n*/(*n*−1)’ to adjust for sample size facilitated cross-population comparability. *H*_E_ was averaged across the 9 loci in each population to provide a measure of population diversity.

Using infection haplotypes, multi-locus linkage disequilibrium (LD) was assessed by the standardised index of association (*I*_A_^S^) using the web-based LIAN 3.5 software [24]. Testing the null hypothesis of linkage equilibrium, the significance of the *I*_A_^S^ estimates was assessed using 10,000 random permutations of the data. Only isolates with no missing data at any of the loci investigated could be included in this analysis.

The differentiation between pairs of populations was measured using the pairwise *F*_ST_ metric in the FSTAT software version 2.9.3, with Bonferroni correction for multiple tests, and a significance level of 0.05. Pairwise tests were undertaken for 1) Kota Kinabalu district versus Kota Marudu district, and 2) Kudat Division versus West Coast Division. Only the predominant allele at each locus in each isolate was used.

STRUCTURE software version 2.3.3 [25] was used to determine the most likely number of populations (*K*) in the total sample, and derive the probability of ancestry of each isolate to each of the *K* populations. The software required infection haplotypes and tolerated missing data. For optimal performance, samples missing data at 3 or more loci were excluded. Model parameters were admixture with correlated allele frequencies. Twenty replicates, with 100,000 burnins and 100,000 iterations were run for each of *K* from 1–10. The most probable *K* was derived by calculating Δ*K* as described elsewhere [26] for each of *K* = 2–9. STRUCTURE results were displayed in barplots using *distruct* software version 1.1 [27].

The genetic relatedness between sample pairs was assessed by a simple measure of the proportion of alleles shared between haplotype pairs (ps), and using (1-ps) as a measure of genetic distance [28]. Samples with missing data at one or more loci were excluded from analysis. An unrooted neighbour-joining tree [29] was generated from the distance matrix using the APE (Analysis of Phylogenetics and Evolution) package in R [30], [31].

With the exception of the neighbour-joining tree, recurrent infections (potentially non-independent) were excluded from the population genetic analyses.

Analysis of LD, population differentiation, structure, and genetic relatedness, were all undertaken with and without the msp1F3 locus. Exclusion of the locus had negligible impact on the results (same as test results excluding other, putatively neutral, loci) and, thus, the results are presented with inclusion of the msp1F3 data.

Temporal analysis of haplotype persistence was conducted on samples collected over a 29 months period from September 2011 to January 2013, in which all non-recurrent (i.e. independent isolates) were included with the exception of those from Tawau, which were detected by active surveillance over a shorter period of time.

## Results

### Samples and Genotyping

A total of 97 *P. vivax* samples collected between September 2010 and January 2013 were included in the study (summary in Table 1), largely representing individuals residing in Kudat Division (*n* = 40, 2 in recurrent infections) or West Coast Division (*n* = 40, 6 in recurrent infections). Additional *P. vivax* samples collected from the Tawau (*n* = 12), Sandakan (*n* = 3) and Interior (*n* = 2) divisions were too small to gauge local diversity patterns and were thus included only in the Sabah-wide measures. Overall 98% (93/97) of the isolates exhibited successful genotype calls at 7–9 loci. The remaining 4 isolates exhibited ≥5 successful genotype calls.

**Table 1:** Sample Details.

Division	District	Sample size	Age Range (years)
Kudat	**Kota Marudu**	**24**	
	Kudat	9 (1 recurrent)	
	Pitas	7 (1 recurrent)	
	**Total**	**40 (2 recurrent)**	**4**–**79 (median 23)**
West Coast	**Kota Kinabalu**	**25 (4 recurrent)**	
	Tuaran	6 (2 recurrent)	
	Pulau Gaya	4	
	Ranau	2	
	Penampang	2	
	Papar	1	
	**Total**	**40 (6 recurrent)**	**13**–**54 (median 24)**
Tawau	Tawau	12	
Sandakan	Sandakan	3	
Interior	Sipitang	2	
	1 **All Sabah**	**97 (8 recurrent)**	**4**–**79 (median 24)**

^1^ Including 2 samples from the Interior Division and 3 samples from Sandakan Division.

### Within-host and Population Diversity

Across Sabah, the prevalence of polyclonal infections was 25.8% (23/89; Table 2) and a large proportion of these infections (60.9% [14/23]) exhibited multiple alleles at just one of the 9 loci investigated, indicative of highly related clones (Figure S1). The prevalence of polyclonal infections in each of the divisions and districts remained below 30% (Table 2). On the basis of the apparent diversity of each of the 9 markers, the majority of polyclonal infections (>90%) would have been detected with the 6 most diverse markers, and 100% of polyclonal infections would have been detected with the 8 most diverse markers (Figure S2). In accordance with the modest prevalence of polyclonal infections, infection complexity, as measured by the MOI, was modest in the Sabah-wide sample set (mean MOI  = 1.30), as well as at the division (mean MOI  = 1.37 in Kudat and 1.21 in the West Coast Division), and district levels (mean MOI  = 1.38 in Kota Marudu and 1.19 in Kota Kinabalu) (Table 2).

**Table 2:** Within-host and Population Diversity.

Population	% Polyclonal Infections	Average MOI (range)	Population Diversity (mean *H*_E_ ± SE [range])
Kota Marudu	29.2% (7/24)	1.38 (1–4)	0.583±0.041 (0.333–0.706)
Kota Kinabalu	23.8% (5/21)	1.19 (1–2)	0.667±0.013 (0.595–0.733)
Kudat Division	29.0% (11/38)	1.37 (1–4)	0.744±0.025 (0.617–0.842)
West Coast Division	17.6% (6/34)	1.21 (1–3)	0.763±0.021 (0.668–0.825)
All Sabah	25.8% (23/89)	1.30 (1–4)	0.825±0.021 (0.723–0.916)

Population diversity was moderate to high, with expected heterozygosity (*H*_E_) reaching 0.825 Sabah wide, remaining high at the divisional level (Kudat, *H*_E_  = 0.744; West Coast Division, *H*_E_  = 0.763), but slightly lower at the district level (Kota Kinabalu *H*_E_  = 0.667; Kota Marudu *H*_E_  = 0.583) (Table 2).

### Population Structure and Differentiation

Pair-wise measures of the fixation index revealed significant differentiation between Kudat and the West Coast Division (*F*_ST_  = 0.125), this differentiation being more apparent at the district level between Kota Marudu and Kota Kinabalu (*F*_ST_  = 0.243) (Table 3).

**Table 3:** Pair-wise differentiation.

District	Kota Marudu
Kota Kinabalu	0.243*
Division	Kudat Division
West Coast Division	0.125*

*F*_ST_ values. Indicative adjusted nominal level (5%) for multiple comparisons is 0.025 (*significant after correction). Pair-wise

**Figure 2:**
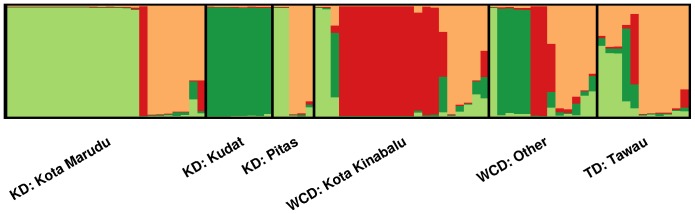
Population structure inferred by STRUCTURE software at K = 4. Results presented with demarcations by district. KD  =  Kudat Division, WCD  =  West Coast Division, TD  =  Tawau Division. Each vertical bar represents an individual sample and each colour represents one of the 4 clusters (sub-populations) defined by STRUCTURE. For each sample, the predicted ancestry to each of the 4 sub-populations is represented by the colour-coded bars. K1  =  light green, K2  =  dark green, K3  =  red, K4  =  orange. Within each demarcation, samples are ordered according to their ancestral proportions from K1 to K4. Some isolates display predominant ancestry to a single sub-population (vertical lines with a single colour), and others appear to display ancestry to more than one sub-population (vertical lines split into multiple colour blocks).

STRUCTURE analysis on the Sabah-wide dataset demonstrated multiple sub-populations. The most likely number of sub-populations was estimated to be four (Figure S3). None of these sub-populations were observed exclusively in a given district, but the representation of three of the sub-populations (K1, K2 and K3) appeared to be moderately disproportionate between certain districts (Figure 2). As demonstrated by the pair-wise *F*_ST_, Kota Marudu and Kota Kinabalu exhibited moderately different STRUCTURE profiles. In Kota Marudu, 66.7% (16/24) of isolates demonstrated predominant ancestry (>75%) to the K1 sub-population, in contrast to 9.5% (2/21) of isolates from Kota Kinabalu. The major sub-population in Kota Kinabalu was K3, with 57.1% (12/21) of isolates demonstrating predominant ancestry, relative to 4.2% (1/24) in Kota Marudu. Although sample size was small, in Kudat district the K2 isolate predominated (100% [8/8]).

**Figure 3:**
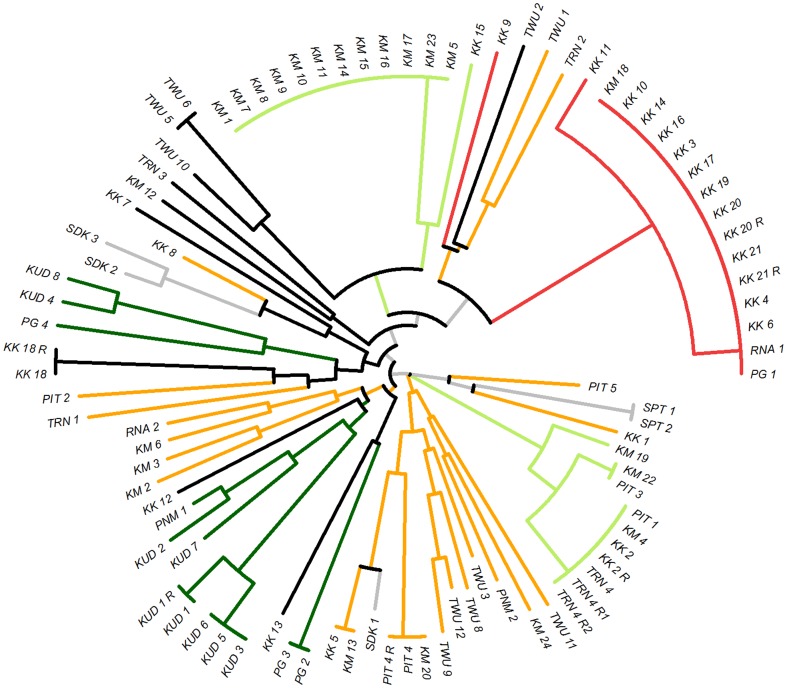
Unrooted neighbour-joining trees illustrating genetic relatedness between isolates. Tree generated with data from 92 isolates with no missing data at 6 loci. Branches are colour-coded by STRUCTURE clusters at K = 4 (see Figure 3). K1  =  light green, K2  =  dark green, K3  =  red, K4  =  orange. Black  =  samples with a maximum ancestry to any of the 4 clusters <75%. Grey  =  samples from the Sandakan or Interior Divisions (not included in the STRUCTURE analysis). KK  =  Kota Kinabalu, KM  =  Kota Marudu, KUD  =  Kudat, PG  =  Pulau Gaya, PIT  =  Pitas, PNM  =  Penampang, SDK  =  Sandakan, SPT  =  Sipitang, TRN  =  Tuaran, TWU  =  Tawau, RNA  =  Ranau. R  =  recurrent infection.

A neighbour-joining tree based on a pairwise distance matrix at 6 loci (MS10, MS8 and MS20 excluded) was generated from all samples with no missing data at these loci (*n* = 92) (Figure 3). The subset of 6 loci was selected to optimize sample size and geographic spread as well as marker number as the distance matrix analysis does not permit missing data. The impact of different marker subsets was investigated, and no notable alterations to the general clustering pattern of the tree were observed (data not presented). The neighbour-joining tree enabled further visual assessment of the population structure and genetic relatedness amongst the Sabahan samples. A large cluster of isolates, predominantly from Kota Kinabalu, which appeared to reflect the K3 sub-population, demonstrated identical or near-identical haplotypes. The K1 sub-population appeared to be split into one large cluster of identical isolates, almost exclusively from Kota Marudu, and a second, cluster of identical or near-identical isolates derived from a range of districts spanning the Kudat and West Coast Divisions. The two clusters were not highly related to one another but possibly diverged from a recent common ancestor, with a recent recombination event breaking down the proportion of shared alleles. Aside from the large clonal expansions of K1 and K3, throughout the sample set, smaller groups of isolates with identical or near-identical haplotypes were observed, interspersed with a range of less closely related isolates reflecting the K4 sub-population.

### Linkage Disequilibrium

When all samples were included in the analysis, high levels of linkage disequilibrium (LD) were observed Sabah-wide and at the division and district levels (*I*_A_^S^ range 0.320 to 0.690; all *P*<0.0001) (Table 4). However, when each unique haplotype was represented only once, marked reductions in LD were observed in all of the datasets (*I*_A_^S^ range -0.003 to 0.109), with loss of significance in the West Coast division (*P* = 0.283) and Kota Kinabalu (*P* = 0.606).

**Table 4:** Linkage Disequilibrium.

	1All Samples			Unique Haplotypes		
Population	*N*	*I*_A_^S^	*P-value*	*N*	*I*_A_^S^	*P-value*
Kota Marudu	20	0.515	*P*<0.0001	10	0.073	*P* = 0.0076
Kota Kinabalu	19	0.690	*P*<0.0001	9	-0.003	*P* = 0.6060
Kudat Division	31	0.410	*P*<0.0001	18	0.109	*P*<0.0001
West Coast Division	30	0.543	*P*<0.0001	17	0.007	*P* = 0.2830
All Sabah	70	0.320	*P*<0.0001	39	0.048	*P*<0.0001

^1^ All samples with no missing data at 9 loci.

### Temporal Haplotype Dynamics

**Figure 4:**
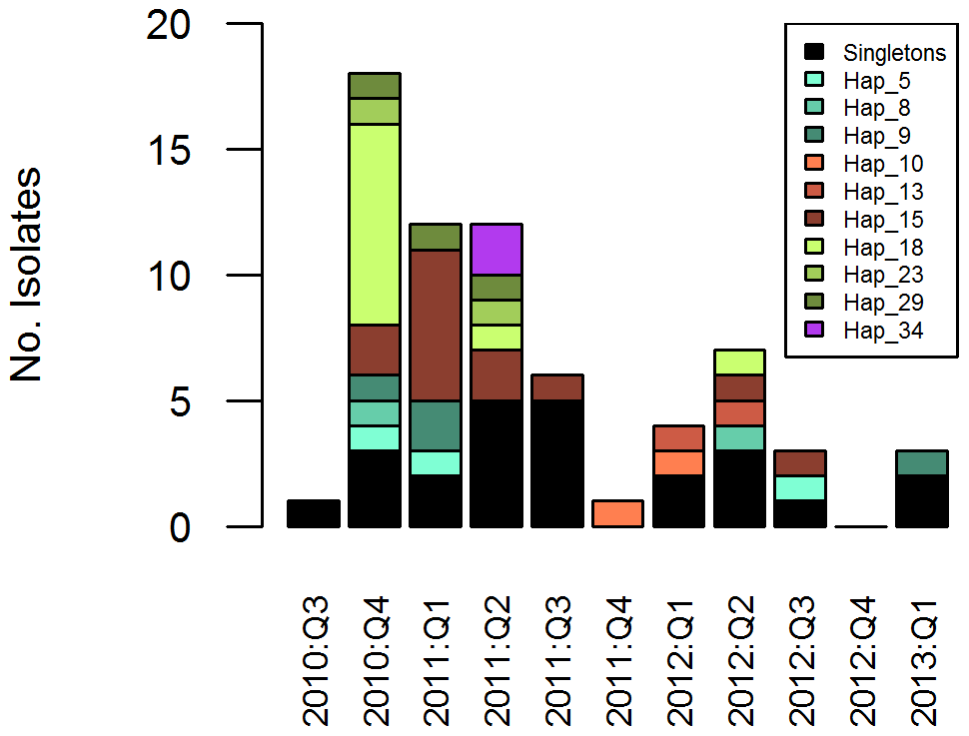
Temporal haplotype dynamics in independent samples. Recurrent samples excluded. Dates split by yearly quarters. Note, 2010 Q3 and 2013 Q1 only comprise samples collected in September and January, respectively. Haplotypes with a minimum of 8 identical alleles at the 9 loci investigated and belonging to the same STRUCTURE sub-population (at K = 4) were grouped together as a single haplotype [22]. Singleton haplotypes were grouped together for simple visual representation in the plot (black bars).

A total of 34 distinct haplotypes were observed in 72 samples collected between September 2011 and January 2013. A high frequency of these haplotypes (29.4% [10/34]) were observed more than once in independent samples, the frequency of multiple incidences ranging from 2–13 (Figure 4). The most frequently observed haplotypes were haplotype 15 (13 incidences), corresponding to the STRUCTURE-defined K3 cluster observed predominantly in Kota Kinabalu, and haplotype 18 (10 incidences), corresponding to the K1 cluster observed predominantly in Kota Marudu. Over the duration of the study, haplotype 15 was first observed in the last quarter of 2010 (one incidence in Kota Marudu and one in Kota Kinabalu in December 2012), peaking in the first quarter of 2011 (5 incidences in Kota Kinabalu with presentation ranging from the same day to 66 days apart). This strain persisted with two incidences observed in the second quarter of 2011 in each of Pulau Gaya and Ranau (both West Coast Division), and three further incidences in Kota Kinabalu between the third quarters of 2011 and 2012.

Haplotype 18 was first observed in the last quarter of 2010, with a peak of 8 incidences, all observed in Kota Marudu, and ranging from presentation on the same day to 65 days apart. Fewer incidences of haplotype 18 were detected in 2011 and 2012, with individual presentations observed in Kota Marudu in the second quarters of these respective years.

The other multiple incidence haplotypes observed in the duration of the study generally presented at more distal time points, all observed at frequencies of 3 or less within a given 3 month period. Within the duration of the study period assessed (September 2011 to January 2013), the longest detected persistence of the same haplotype (haplotype 9) was 796 days. As observed with several other haplotypes, haplotype 9 was detected in a range of geographic locations, appearing first in Tuaran (West Coast Division), followed by Kota Kinabalu, Kota Marudu, and then Pitas.

### Recurrent Infections

During the study period, 7 patients presented with recurrent *P. vivax* infections (one patient twice, all others once) between 28 and 104 days following primary therapy (Table 5). *P. vivax* parasitaemia was not detected by microscopy at 3 of the 8 ‘primary’ presentations and thus primaquine (PQ) radical therapy was not prescribed for these cases. All sample pairs demonstrated homologous genotype calls at all of the successfully genotyped loci. The recurrences were sourced from individuals residing in a range of districts, and exhibited a range of haplotypes, including three haplotypes (9, 15 and 22) that had been observed in more than one independent (i.e. non-recurrent) infection (Figure 4).

**Table 5:** Infection haplotypes in patients with recurrent *P. vivax* infections.

1Sample	PQ therapy	Interval	Year: Quarter	Haplotype	Marker								
		(days)			3.27	MS1	MS10	MS12	MS16	MS20	MS5	MS8	msp1F3
KUD_1	Yes	-	2010:Q3	2NA	317	233	Fail	208	428	206	173	307	255
KUD_1_R	Yes	92	2010:Q4	2NA	317	233	Fail	208	428	206	173	307	255
PIT_4	No	-	2010:Q4	22	312	227	195	211	475	200	179	231	345
PIT_4_R	Yes	40	2010:Q4	22	312	227	195	211	475	200	179	231	345
KK_2	Yes	-	2011:Q1	9	271	227	186	202	446	194	179	280	261
KK_2_R	Yes	104	2011:Q2	9	271	227	186	202	446	194	179	280	261
KK_18	No	-	2010:Q4	2NA	283	230	186	Fail	465	203	170	263	255
KK_18_R	Yes	46	2011:Q1	2NA	283	230	186	179	465	203	170	263	255
KK_20	No	-	2011:Q1	15	287	224	177	205	452	203	182	237	325
KK_20_R	Yes	85	2011:Q2	15	287	224	177	205	452	203	182	237	325
KK_21	Yes	-	2011:Q1	15	287	224	177	205	452	203	182	237	325
KK_21_R	Yes	53	2011:Q1	15	287	224	177	205	452	203	182	237	325
TRN_4	Yes	-	2010:Q4	9	271	227	186	202	446	194	179	280	261
TRN_4_R1	Yes	28	2010:Q4	9	271	227	186	202	446	194	179	280	261
TRN_4_R2	Yes	84	2011:Q2	2NA	271	227	186	Fail	446	Fail	Fail	280	261

=  Kudat (Kudat Division), PIT  =  Pitas (Kudat Division), KK  =  Kota Kinabalu (West Coast Division), TRN  =  Tuaran (West Coast Division), R  =  Recurrent.^1^ KUD

^2^ Not assigned a haplotype owing to missing data.

## Discussion

Effective reduction in local malaria transmission can lead to complex parasite transmission dynamics, with small populations being more effected by the impact of migration and random genetic drift [32]. The consequences of these processes, including the risks of epidemic expansions, and enhanced vulnerability to the emergence of drug resistant strains have clear implications for malaria elimination [33]. We investigated the population structure and transmission dynamics in *P. vivax* isolates sourced largely from the Kudat and West Coast divisions of Sabah. These two divisions account for approximately 36% of Sabah's population. As malaria elimination approaches, our results demonstrate that the low level of local transmission is associated with frequent clonal expansions, possibly resulting from the presentation of new parasite strains introduced by migration or generated locally by mutation or recombination. Although the incidence of these strains appeared to decline over time, several strains were detected in the population more than a year after the initial expansion, possibly reflecting long-term persistence. The available evidence indicated that, amongst other factors, homologous infections which we hypothesise to be relapses might facilitate the persistence of these strains in the local population.

Inevitable sample size constraints in pre-elimination settings present significant analytical challenges. In 2011, only 628 notifications of *P. vivax* were recorded across Sabah, with 151 (24%) of these reported from the Kudat and West Coast Divisions [9]. Nonetheless, for the purpose of population genetic characterisation, we were able to capture sufficient sample numbers from two districts, Kota Marudu and Kota Kinabalu, together representing 10% of the reported vivax cases in Sabah in 2011 [9]. Additional samples captured from other districts enabled a rough assessment of the relatedness between *P. vivax* isolates across the state.

Our analyses suggested that for most intents, Sabah should not be considered a single population. Rather, as seen in *P. falciparum*, the *P. vivax* population demonstrated multiple sub-populations [10]. STRUCTURE analysis revealed evidence of four sub-populations amongst the sites investigated. Although none of these sub-populations were exclusive to a given district, Kota Marudu and Kota Kinabalu exhibited moderately different STRUCTURE profiles, largely reflecting different representation of two sub-populations (K1 and K3). The pair-wise fixation index between Kota Marudu and Kota Kinabalu, located just 130 Km apart, demonstrated significant differentiation (*F*_ST_  = 0.243), approaching levels observed in international comparisons [12]. Although these samples represent a large proportion of the *P. vivax* isolates presenting in the Kudat and West Coast Divisions (based on the 2011 notifications described above), we should nonetheless acknowledge that sample size may influence the detection of population structure.

Despite differences in *P. vivax* incidence, similar patterns of diversity were observed in Kota Marudu and Kota Kinabalu, suggesting that a common mechanism other than local transmission had a strong impact in both settings. In both districts, and across Sabah as a whole, rates of polyclonal infection were modest (less than 30%), and a large proportion of these polyclonal infections only displayed multiple alleles at one of the nine loci investigated (∼60%). By genotyping the 6 most diverse (highest *H*_E_) markers, we demonstrated that more than 90% of the polyclonal infections identified with the full set of 9 markers would have been detected. Therefore, we anticipate that genotyping at additional loci would not lead to a major increase in detection of polyclonal infections. Moreover, if extra markers are required to identify a polyclonal infection, by definition, it remains that the clones within that infection are highly related (i.e. overall infection diversity is modest). In accordance with the modest rates of polyclonal infection, the mean MOI was modest in all sites tested (less than 1.5 clones per infection). These measures of within-host diversity are generally lower than those reported for other tropical endemic sites in the Asia-Pacific region [12], [20], [23], [34], [35], and largely indicative of low levels of local transmission, as has been observed in other locations after aggressive malaria control interventions [36].

Despite the modest rates of polyclonal infection, population level diversity remained moderate in both Kota Marudu and Kota Kinabalu, which may reflect frequent introductions of new parasite strains. With small populations generally being more affected by the impact of migration [32], this may present an important reservoir for the introduction of new parasite strains, which may then result in small outbreaks. Neighbour-joining analysis illustrated large clusters of identical or nearly identical isolates in each of Kota Marudu and Kota Kinabalu. When these isolates were represented just once in linkage disequilibrium (LD) analysis, the high levels of LD observed in the presence of all isolates (*I*_A_^S^ >0.5) declined sharply (*I*_A_^S^ ≤0.07). The short temporal span within which peak frequencies of identical strains were observed, and the moderate geographic confinement of these incidences, further supports outbreak dynamics. Sabah's rapidly shrinking *P. vivax* population may thus have significant adverse public health consequences, including a greater impact of migration and increased risk of epidemics. In this low endemic setting, low levels of acquired immunity in the host population may have compounded the risk of epidemics. Indeed, in contrast to high transmission settings such as the island of New Guinea, where the majority of patients presenting with symptomatic *P. vivax* infection are young children [37], in Sabah, the majority of symptomatic patients were adults, with a median age of 24 years (Table 1).

Another public health risk is presented by the persistence of specific strains in the population, with concurrent risks of parasite spread and maintenance of population diversity. In a slightly higher transmission setting in Brazil, only short short-term persistence of parasite lineages (up to 5 months) was observed [22]. In contrast, in Sabah, persistence of certain haplotypes was observed for up to two years, and this may be an underestimation limited by the duration of the study. As suggested by Ferreira and colleagues, variation in the persistence of parasite lineages might reflect a number of factors including differences in parasite mutation, recombination, and migration rates, population size, and acquired variant-specific immunity in the host population [22]. Homologous relapses might support the persistence of certain parasite lineages. Eight pairs of primary and recurrent infections were captured during the study, all representing as homologous haplotypes. The majority of these cases are likely to be relapses. Firstly, the timing of the recurrences (28 to 104 days after primary infection), were in accordance with the expected timing of relapses in tropical parasite strains [38]. Secondly, low local transmission levels in the regions concerned ensured a low probability of re-infection, although the potential impact of transmission hot-spots cannot be excluded [39]. Lastly, in at least 3 cases (where the primary infection was misdiagnosed as a species other than *P. vivax* [7]), adequate anti-relapse therapy was not administered thus increasing the risk of relapse which occurs naturally in this equatorial region in almost 80% of cases [38].

We observed incidences of the same haplotype identified in different districts and divisions at different time points, compatible with a process mediated by relapsing infections. This offers some promise for the ability to trace at least the short-term movements of particular parasite strains, with potential utility for transmission mapping efforts. However, better approaches for identifying recent mutations and recombinants are required. High-throughput genome sequencing efforts should facilitate this objective [40], [41].

In the current study, interspersed amongst the clusters of identical haplotypes, we observed a number of singletons, many exhibiting very different haplotypes from one another. Some of these isolates might reflect remnants of previous expansions following the introduction of new strains which have persisted in the population. The diversity of these isolates largely explains the population diversity observed in Kota Marudu (*H*_E_  = 0.583) and Kota Kinabalu (*H*_E_  = 0.667). It follows that, despite lower *P. vivax* incidence, as a major industrial, administrative and commercial center, the higher level of *P. vivax* diversity observed in Kota Kinabalu might reflect more frequent immigration of novel genotypes. Further transmission mapping efforts over longer periods of time should enable greater insight into the role of migration in maintaining local *P. vivax* diversity.

In conclusion, our study highlights the potential utility of genotyping as a tool to inform *P. vivax* transmission dynamics, population diversity and structure. A better understanding of these dynamics in pre-elimination settings will inform on the underlying processes that sustain the dynamic transmission of parasite populations, thus assisting malaria control programs to prioritise activities and mobilise appropriate resources.

## Supporting Information

Figure S1 **Distribution of number of loci with multiple alleles.** Samples  =  all Sabah excluding recurrent infections (n = 89)
(TIFF)

Figure S2 **Relationship between the number of markers genotyped and the proportion of polyclonal infections identified.** Samples  =  all Sabah polyclonal infections excluding recurrent infections (n = 23). Markers were added consecutively in order of decreasing *H*_E_ as follows: Pv3.27, MS16, msp1F3, MS10, MS20, MS8, MS5, MS1 and MS12. All polyclonal infections were detected with the first 8 markers.
(TIFF)

Figure S3 **Distribution of Delta K (ΔK) against K.** Peak ΔK observed at K = 4.
(TIFF)

## References

[1] Guerra CA, Howes RE, Patil AP, Gething PW, Van Boeckel TP, et al (2010) The international limits and population at risk of Plasmodium vivax transmission in 2009. PLoS Negl Trop Dis 4: e774. 20689816 10.1371/journal.pntd.0000774 PMC2914753

[2] Price RN, Douglas NM, Anstey NM (2009) New developments in Plasmodium vivax malaria: severe disease and the rise of chloroquine resistance. Curr Opin Infect Dis 22: 430–435. 19571748 10.1097/QCO.0b013e32832f14c1

[3] Carlton JM, Sina BJ, Adams JH (2011) Why is Plasmodium vivax a neglected tropical disease? PLoS Negl Trop Dis 5: e1160. 21738804 10.1371/journal.pntd.0001160 PMC3125139

[4] Feachem RG, Phillips AA, Hwang J, Cotter C, Wielgosz B, et al (2010) Shrinking the malaria map: progress and prospects. Lancet 376: 1566–1578. 21035842 10.1016/S0140-6736(10)61270-6 PMC3044848

[5] World Health Organization (2010) World Malaria Report 2010. World Health Organization; Geneva 2010.

[6] Joveen-Neoh WF, Chong KL, Wong CM, Lau TY (2011) Incidence of malaria in the interior division of sabah, malaysian borneo, based on nested PCR. J Parasitol Res 2011: 104284. 22013506 10.1155/2011/104284 PMC3195446

[7] Barber BE, William T, Grigg MJ, Yeo TW, Anstey NM (2013) Limitations of microscopy to differentiate Plasmodium species in a region co-endemic for Plasmodium falciparum, Plasmodium vivax and Plasmodium knowlesi. Malar J 12: 8. 23294844 10.1186/1475-2875-12-8 PMC3544591

[8] Rundi C (2011) Malaria Elimination in Malaysia. Third annual APMEN technical and business meeting, 9–12 May 2011; Kota Kinabalu, Malaysia. Available: http://apmen.org/apmen-iii-meeting-proceedings/. Accessed 2013 Jul 28.

[9] William T, Rahman HA, Jelip J, Ibrahim MY, Menon J, et al (2013) Increasing incidence of Plasmodium knowlesi malaria following control of P. falciparum and P. vivax Malaria in Sabah, Malaysia. PLoS Negl Trop Dis 7: e2026. 23359830 10.1371/journal.pntd.0002026 PMC3554533

[10] Anthony TG, Conway DJ, Cox-Singh J, Matusop A, Ratnam S, et al (2005) Fragmented population structure of plasmodium falciparum in a region of declining endemicity. J Infect Dis 191: 1558–1564. 15809916 10.1086/429338

[11] The Asia Pacific Malaria Elimination Network website. Available: http://apmen.org/. Accessed 2013 Jul 30.

[12] Imwong M, Nair S, Pukrittayakamee S, Sudimack D, Williams JT, et al (2007) Contrasting genetic structure in Plasmodium vivax populations from Asia and South America. Int J Parasitol 37: 1013–1022. 17442318 10.1016/j.ijpara.2007.02.010

[13] Karunaweera ND, Ferreira MU, Hartl DL, Wirth DF (2007) Fourteen polymorphic microsatellite DNA markers for the human malaria parasite Plasmodium vivax. Mol Ecol Notes 7: 172–175.

[14] Koepfli C, Mueller I, Marfurt J, Goroti M, Sie A, et al (2009) Evaluation of Plasmodium vivax genotyping markers for molecular monitoring in clinical trials. J Infect Dis 199: 1074–1080. 19275476 10.1086/597303

[15] Gething PW, Elyazar IR, Moyes CL, Smith DL, Battle KE, et al (2012) A long neglected world malaria map: Plasmodium vivax endemicity in 2010. PLoS Negl Trop Dis 6: e1814. 22970336 10.1371/journal.pntd.0001814 PMC3435256

[16] Department of Statistics, Malaysia (2011) Population Distribution and Basic Demographic Characteristics, Population and Housing Census of Malaysia 2010. Kuala Lumpur: Department of Statistics, Malaysia.

[17] Barber BE, William T, Grigg MJ, Menon J, Auburn S, et al (2013) A prospective comparative study of knowlesi, falciparum, and vivax malaria in Sabah, Malaysia: high proportion with severe disease from Plasmodium knowlesi and Plasmodium vivax but no mortality with early referral and artesunate therapy. Clin Infect Dis 56: 383–397. 23087389 10.1093/cid/cis902

[18] Padley D, Moody AH, Chiodini PL, Saldanha J (2003) Use of a rapid, single-round, multiplex PCR to detect malarial parasites and identify the species present. Ann Trop Med Parasitol 97: 131–137. 12803868 10.1179/000349803125002977

[19] Imwong M, Tanomsing N, Pukrittayakamee S, Day NP, White NJ, et al (2009) Spurious amplification of a Plasmodium vivax small-subunit RNA gene by use of primers currently used to detect P. knowlesi. J Clin Microbiol 47: 4173–4175. 19812279 10.1128/JCM.00811-09 PMC2786678

[20] Karunaweera ND, Ferreira MU, Munasinghe A, Barnwell JW, Collins WE, et al (2008) Extensive microsatellite diversity in the human malaria parasite Plasmodium vivax. Gene 410: 105–112. 18226474 10.1016/j.gene.2007.11.022

[21] Anderson TJ, Su XZ, Bockarie M, Lagog M, Day KP (1999) Twelve microsatellite markers for characterization of Plasmodium falciparum from finger-prick blood samples. Parasitology 119 (Pt 2): 113–125. 10466118 10.1017/s0031182099004552

[22] Ferreira MU, Karunaweera ND, da Silva-Nunes M, da Silva NS, Wirth DF, et al (2007) Population structure and transmission dynamics of Plasmodium vivax in rural Amazonia. J Infect Dis 195: 1218–1226. 17357061 10.1086/512685

[23] Koepfli C, Timinao L, Antao T, Barry AE, Siba P, et al (2013) A Large Reservoir and Little Population Structure in the South Pacific. PLoS One 8: e66041. 23823758 10.1371/journal.pone.0066041 PMC3688846

[24] Haubold B, Hudson RR (2000) LIAN 3.0: detecting linkage disequilibrium in multilocus data. Linkage Analysis. Bioinformatics 16: 847–848. 11108709 10.1093/bioinformatics/16.9.847

[25] Pritchard JK, Stephens M, Donnelly P (2000) Inference of population structure using multilocus genotype data. Genetics 155: 945–959. 10835412 10.1093/genetics/155.2.945 PMC1461096

[26] Evanno G, Regnaut S, Goudet J (2005) Detecting the number of clusters of individuals using the software STRUCTURE: a simulation study. Mol Ecol 14: 2611–2620. 15969739 10.1111/j.1365-294X.2005.02553.x

[27] Rosenberg NA (2004) *Distruct*: a program for the graphical display of population structure. Molecular Ecology Notes 4: 137–138.

[28] Bowcock AM, Ruiz-Linares A, Tomfohrde J, Minch E, Kidd JR, et al (1994) High resolution of human evolutionary trees with polymorphic microsatellites. Nature 368: 455–457. 7510853 10.1038/368455a0

[29] Saitou N, Nei M (1987) The neighbor-joining method: a new method for reconstructing phylogenetic trees. Mol Biol Evol 4: 406–425. 3447015 10.1093/oxfordjournals.molbev.a040454

[30] Ihaka R, Gentleman R (1996) R: A language for data analysis and graphics. Journal of Computational and Graphical Statistics 5: 299–314.

[31] Paradis E, Claude J, Strimmer K (2004) APE: Analyses of Phylogenetics and Evolution in R language. Bioinformatics 20: 289–290. 14734327 10.1093/bioinformatics/btg412

[32] Travis J (1990) The interplay of population dynamics and the evolutionary process. Philos Trans R Soc Lond B Biol Sci 330: 253–259. 1981622 10.1098/rstb.1990.0196

[33] Miotto O, Almagro-Garcia J, Manske M, Macinnis B, Campino S, et al (2013) Multiple populations of artemisinin-resistant Plasmodium falciparum in Cambodia. Nat Genet 45: 648–655. 23624527 10.1038/ng.2624 PMC3807790

[34] Gunawardena S, Karunaweera ND, Ferreira MU, Phone-Kyaw M, Pollack RJ, et al (2010) Geographic structure of Plasmodium vivax: microsatellite analysis of parasite populations from Sri Lanka, Myanmar, and Ethiopia. Am J Trop Med Hyg 82: 235–242. 20133999 10.4269/ajtmh.2010.09-0588 PMC2813164

[35] Van den Eede P, Erhart A, Van der Auwera G, Van Overmeir C, Thang ND, et al (2010) High complexity of Plasmodium vivax infections in symptomatic patients from a rural community in central Vietnam detected by microsatellite genotyping. Am J Trop Med Hyg 82: 223–227. 20133996 10.4269/ajtmh.2010.09-0458 PMC2813161

[36] Van den Eede P, Van der Auwera G, Delgado C, Huyse T, Soto-Calle VE, et al (2010) Multilocus genotyping reveals high heterogeneity and strong local population structure of the Plasmodium vivax population in the Peruvian Amazon. Malar J 9: 151. 20525233 10.1186/1475-2875-9-151 PMC2898784

[37] Mueller I, Galinski MR, Tsuboi T, Arevalo-Herrera M, Collins WE, et al (2013) Natural acquisition of immunity to Plasmodium vivax: epidemiological observations and potential targets. Adv Parasitol 81: 77–131. 23384622 10.1016/B978-0-12-407826-0.00003-5

[38] White NJ (2011) Determinants of relapse periodicity in Plasmodium vivax malaria. Malar J 10: 297. 21989376 10.1186/1475-2875-10-297 PMC3228849

[39] Bousema T, Griffin JT, Sauerwein RW, Smith DL, Churcher TS, et al (2012) Hitting hotspots: spatial targeting of malaria for control and elimination. PLoS Med 9: e1001165. 22303287 10.1371/journal.pmed.1001165 PMC3269430

[40] Auburn S, Marfurt J, Maslen G, Campino S, Ruano Rubio V, et al (2013) Effective preparation of Plasmodium vivax field isolates for high-throughput whole genome sequencing. PLoS One 8: e53160. 23308154 10.1371/journal.pone.0053160 PMC3537768

[41] Chan ER, Menard D, David PH, Ratsimbasoa A, Kim S, et al (2012) Whole genome sequencing of field isolates provides robust characterization of genetic diversity in Plasmodium vivax. PLoS Negl Trop Dis 6: e1811. 22970335 10.1371/journal.pntd.0001811 PMC3435244

