# Supplementary figures and images for: Plasmodium vivax Population Structure and Transmission Dynamics in Sabah Malaysia
Source: PLoS One. 2013 Dec 17;8(12):e82553. doi: 10.1371/journal.pone.0082553 (PMC3866266; doi:10.1371/journal.pone.0082553)

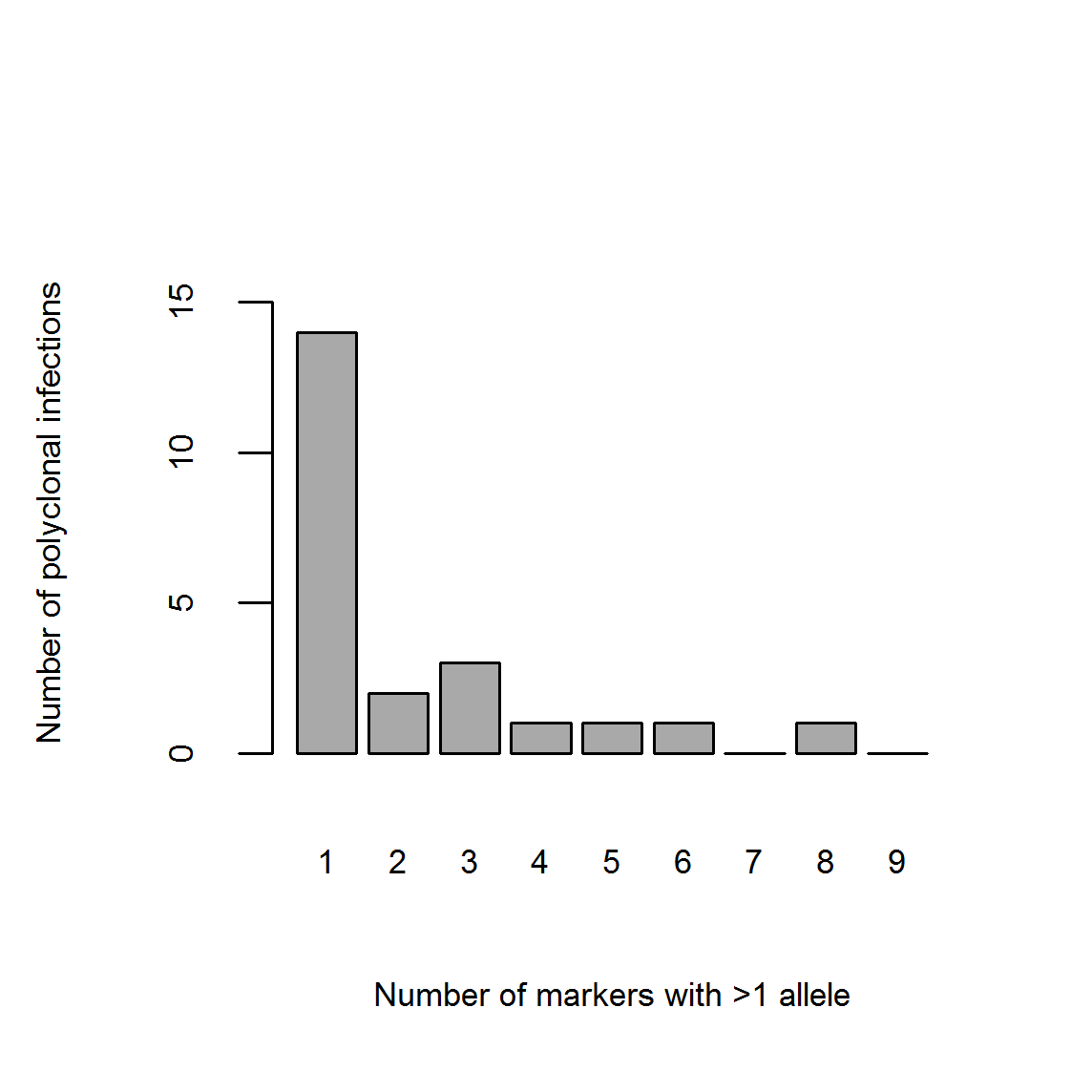

Supplement: Figure S1 — Distribution of number of loci with multiple alleles. Samples = all Sabah excluding recurrent infections (n = 89) (TIFF) [file pone.0082553.s001.tiff]

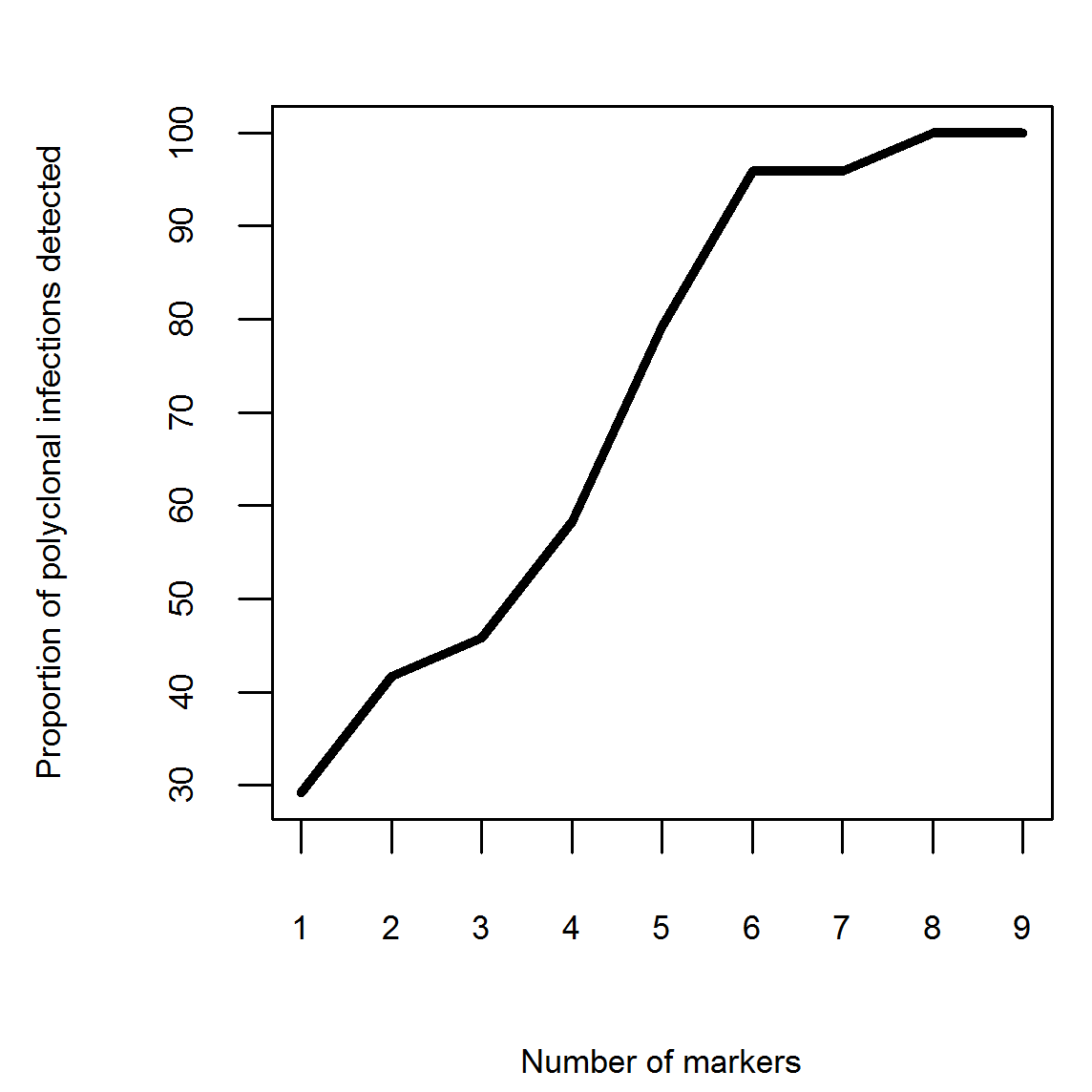

Supplement: Figure S2 — Relationship between the number of markers genotyped and the proportion of polyclonal infections identified. Samples = all Sabah polyclonal infections excluding recurrent infections (n = 23). Markers were added consecutively in order of decreasing H E as follows: Pv3.27, MS16, msp1F3, MS10, MS20, MS8, MS5, MS1 and MS12. All polyclonal infections were detected with the first 8 markers. (TIFF) [file pone.0082553.s002.tiff]

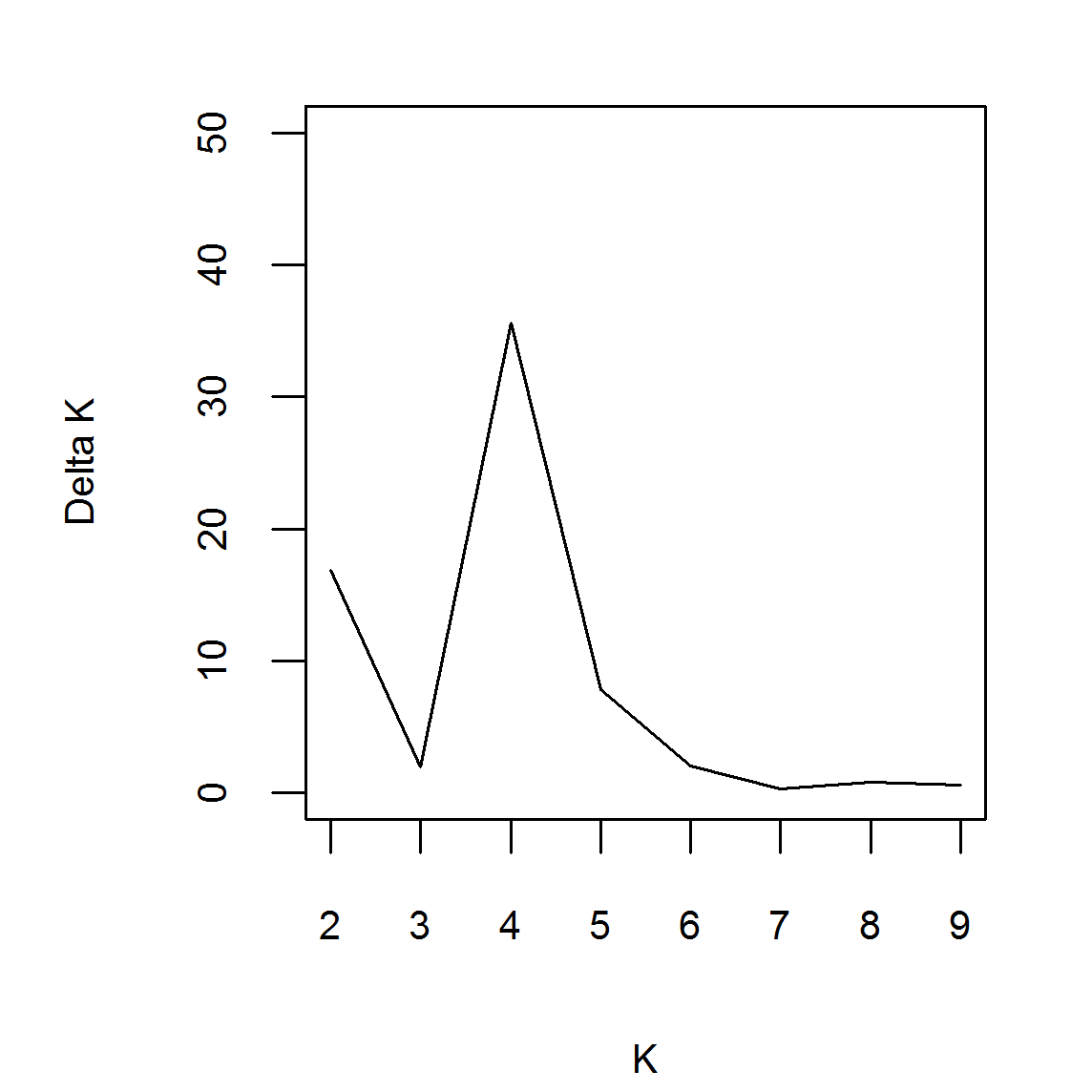

Supplement: Figure S3 — Distribution of Delta K (ΔK) against K. Peak ΔK observed at K = 4. (TIFF) [file pone.0082553.s003.tiff]
